# Comparison of Morphological and Digital-Assisted Analysis for BCL6 Endometrial Expression in Women with Endometriosis

**DOI:** 10.3390/jcm11206164

**Published:** 2022-10-19

**Authors:** Marlyne Squatrito, Silvia Blacher, Laurie Henry, Soraya Labied, Agnès Noel, Michelle Nisolle, Carine Munaut

**Affiliations:** 1 Laboratory of Tumor and Development Biology, GIGA-Cancer, University of Liège, 4000 Liège, Belgium; 2 Obstetrics and Gynecology Department, University of Liege, 4000 Liège, Belgium

**Keywords:** B-cell lymphoma 6, endometriosis, infertility, diagnosis, HSCORE, computer-assisted analysis

## Abstract

BCL6 (B-cell lymphoma 6) is a proto-oncogene and transcriptional repressor initially described as being involved in B-cell lymphoma. Recently, this factor has been identified as a promising tissue biomarker which could be used to diagnose women affected by endometriosis. Previous studies used HSCORE for BCL6 staining quantification in the endometrium. However, this semi-quantitative technique of analysis has some limitations, including a lack of objectivity, robustness, and reproducibility that may lead to intra- and inter-observer variability. Our main goal was to develop an original computer-assisted method to quantify BCL6 staining from whole-slide images reliably. In order to test the efficiency of our new digital method of quantification, we compared endometrial BCL6 expression between fertile and infertile women without or with different stages of endometriosis by using the widely used HSCORE analysis and our new automatic digital image analysis. We find a higher expression of BCL6 in the endometrium of infertile women with endometriosis and women with stage IV endometriosis. Furthermore, we demonstrate a significant correlation between the two types of independent measurements, indicating the robustness of results and also the reliability of our computer-assisted method for BCL6 quantification. In conclusion, our work, by using this original computer-assisted method, enables BCL6 quantification more objectively, reliably, robustly, and promptly compared to HSCORE analysis.

## 1. Introduction

Initially described as a proto-oncogene and transcriptional repressor implicated in the pathogenesis of B-cell lymphoma, BCL6 has recently been identified as a promising tissular biomarker which could be used to diagnose women affected by endometriosis [1,2,3,4]. The recent identification of actors acting in conjunction with BCL6, and the delineation of several target genes have helped to better determine its biological functions [5]. BCL6 contributes to cell cycle control and differentiation, as well as apoptosis inhibition [6,7,8]. The underlying pathophysiological mechanisms leading to endometriosis, a gynecological disease defined by the presence of endometrial tissue outside of the uterine cavity, also include proliferation, apoptosis, autophagy, migration, invasion, fibrosis, angiogenesis, oxidative stress, inflammation, and immune escape [9,10,11].

This disorder is mainly associated with both pelvic pain and infertility and affects about 10–15% of reproductive-age women [12]. Endometriosis is most often classified into four stages according to revised criteria described by the American Society of Reproductive Medicine (rASRM). To define the disease stage, a score taking into account the size of the lesions, their location, and the extent of the adhesions is used [9,13,14]. However, no association between the severity of symptoms and this stage has been shown [15]. The current time span between the onset of women’s perceived symptoms and diagnosis is approximately 8–12 years [16]. Indeed, the range of symptoms can be confusing for the patient and care provider [17,18,19]. This lack of early diagnosis has many consequences for patient health since they are not treated for endometriosis during this period. A late diagnosis, sometimes at an advanced stage of the disease, can already represent a loss of chance for the patient regarding her fertility. Endometriosis, as well as its surgical management, can compromise the chances of pregnancy, particularly by altering the ovarian reserve. This is why an early diagnosis, which may allow oocyte vitrification to be carried out in order to preserve the patient’s chances of fertility, is essential in the management of these women [20]. In addition to the physical consequences, endometriosis induces a deleterious effect on the psychosocial and sexual wellbeing of women and imposes a substantial economic burden from a personal and societal point of view [21,22].

Currently, to reliably diagnose endometriosis, laparoscopy with inspection of the abdominal cavity and histological confirmation of the suspicious lesion must be performed. While alternative methods such as medical imaging, genetic tests, or biomarkers are aimed at alleviating invasive diagnosis, none of them can adequately substitute for surgery at present [23,24,25,26,27]. Recent studies have identified a promising tissular biomarker called B-cell lymphoma 6 (BCL6), which could be used to diagnose women affected by endometriosis [1,2,3,4].

Indeed, Evans-Hoeker et al. examined BCL6 expression in the human eutopic endometrium across the menstrual cycle in women with and without endometriosis and found that BCL6 was overexpressed in the eutopic endometrium of patients with endometriosis, especially during the secretory phase of the menstrual cycle [1]. BCL6 is a promising candidate as a unique diagnostic tissular biomarker for the detection of endometriosis in women. On the contrary, use of BCL6 as a protein biomarker in bodily fluids (such as serum and urine) for a less invasive diagnosis of endometriosis is not appropriate [2]. Furthermore, this factor was also overexpressed in the endometrium of women who suffer from unexplained infertility, recurrent miscarriages, or implantation failure during in vitro fertilization cycles [28,29,30]. These observations prompted researchers to investigate the potential role of BCL6 in the pathophysiological process of endometriosis, especially in infertility [30,31].

All the previous studies describing BCL6 expression in the endometrium of women with or without proven endometriosis use HSCORE for the quantification of BCL6 staining [1,2,3,4]. Evans-Hoeker et al. established an immunostaining cutoff to better discriminate normal versus elevated BCL6 expression [1]. Manual evaluation by an experienced researcher remains a time-consuming and subjective procedure that might lead to intra- and inter-observer variability. The main objective of this study was to compare BCL6 expression in human eutopic endometrium samples by using digital image analysis to obtain objective and highly reproducible quantification from whole-slide images with an adequate dynamic range and the previously widely used HSCORE.

## 2. Materials and Methods

### 2.1. Human Endometrial Tissue Samples

The human endometrial samples were collected from two cohorts of patients recruited in the Gynecology-Obstetrics Department and the Center for Medically Assisted Procreation of the University of Liège (Citadelle Hospital). The first population included endometrium samples obtained from women who underwent laparoscopy for suspicion of endometriosis. The second one included eutopic endometrium samples from infertile women (associated or not with endometriosis). In the first population, the presence of the disease was confirmed by clinicians at the time of laparoscopy in the endometriosis group and classified according to the revised American Society of Reproductive Medicine (ASRM) [14,32]. Women without endometriosis at laparoscopy constituted the control group. All the endometria analyzed were in the secretory phase as determined by the pathologist. The first cohort was further divided into 3 groups: the control fertile group (*n* = 11), the stage I endometriosis group (*n* = 6), and the stage IV endometriosis group (*n* = 10) (subdivided into fertile (*n* = 5) and infertile group (*n* = 5)) (Table 1).

The second cohort was divided into 2 groups: the control infertile group (*n* = 29) and the laparoscopically previously proven endometriosis-infertile group (*n* = 17) (Table 2).

### 2.2. Immunofluorescence Analyses

Endometrial samples fixed in 4% formaldehyde were paraffin-embedded and serially sectioned (5 µm sections). Sections were deparaffinized and rehydrated before endogenous peroxidase activity was blocked by incubating the sections in 3% hydrogen peroxide for 20 min at room temperature (RT). Nonspecific binding sites were blocked by incubation in the “Free Blocking Solution” (Cell Signaling, Danvers, USA) for 1 h at RT. The primary antibody was diluted at 1:100 in the “Dako diluent” (Dako, Jena, Germany) and incubated overnight at 4 °C (rabbit monoclonal anti-BCL6 (Cell Signaling 89369, Danvers, MA, USA)), followed by incubation with the secondary antibody HRP (ENVISION/HRP ready to use, Dako, Glostrup, Denmark) for 30 min at RT. The reaction was revealed using TSA-FITC (Akoya Biosciences, Marlborough, Massachusetts, USA), and the sections were mounted with DAPI FluoromountG mounting medium (SouthernBiotech, Birmingham, AL, USA).

### 2.3. Quantification of Immunostaining

The slides were scanned at 20× magnification using a high-resolution digital slide scanner (NanoZoomer 2.0HT (Hamamatsu Photonics, K.K., Hamamastsu, Japan)). The semi-quantitative analysis of the nuclear labeling of BCL6 was performed using HSCORE (0–4), calculated using the following equation: HSCORE = ∑ Pi (i + 1)/100, with i = the intensity of fluorescence with values of 1, 2, or 3 (weak, moderate, or intense, respectively), and Pi = the percentage of epithelial cells labeled for each intensity, which can range from 0 to 100%. The use of HSCORE is a semi-quantitative immunohistological analysis method that has been previously validated [33]. The HSCORE analyses were performed blinded by two different observers.

**Figure 1:**
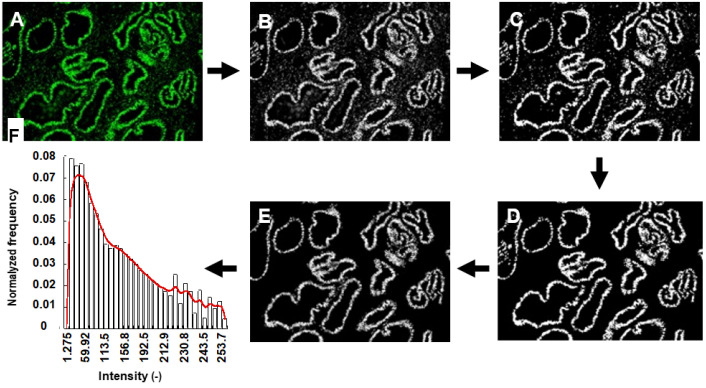
Image processing workflow to quantify epithelial endometrial BCL6 expression: (**A**) original image of one isolated region from the whole scanned image registered in RGB space, (**B**) grey level green component of the RGB image (**C**) automatic thresholding to extract all BCL6 expression (green labeling in stromal and epithelial compartments), (**D**) resulting binary image after filtering stromal cells, (**D**) visualization of the resulting image in 256 gray levels) after background elimination by multiplying the binary image with the grey level green component, (**E**) resulting intensity distribution, (**F**) Histogram of intensities.

Scanned slides were also analyzed with computer-assisted image analysis. In this case, image processing and automatic quantification of BCL6 expression in the epithelial compartment of the endometrium were performed using the image analysis toolbox of MATLAB R2021b according to the following steps: The original images were registered in the full-color red, green, and blue (RGB) space where BCL6 labeling appeared in green and DAPI nuclear staining in blue. Both in the original color image (Figure 1A) as well as in its grey-level green-component (Figure 1B), both epithelial and stromal cells are stained. All cell types appear clearly when an automatic thresholding technique is used to extract the green stained regions (Figure 1C) [34]. The criterion used to discriminate between glandular epithelial cells and stromal cells is their different sizes and morphology. Indeed, as stromal cells appear as small and isolated objects, glandular epithelial cells are connected together as ring-like structures of greater size. Taking into account these differences, morphological filters were applied to the binary images (erosion and open transformations of appropriate sizes) followed by a geodesic reconstruction (Figure 1D) [35]. Finally, the resulting binary image was then multiplied by the grey level green component in order to eliminate the background (Figure 1E). By this process, BCL6 intensities were visualized in 256 grey levels. Eventually, a histogram of intensities was calculated (Figure 1E,F). It must be noticed that the filtering process is strongly dependent on the quality of the images. Indeed, “noise-characteristics” depend on the technical process (tissue fixation, immunofluorescence staining, microscopy-image registration, etc.). For this reason, filtering techniques must be adapted to the particular situation.

The whole methodological process was applied to sections containing at least 20 glands where the intensity distribution was measured simultaneously. A mean intensity distribution of sections corresponding to the patients within the same group was then calculated point by point and in order to produce underling distributions, a smooth operator was applied on histograms Meg Noah (2022). Smooth Operator (Stineman 1D Interpolation) (https://www.mathworks.com/matlabcentral/fileexchange/104195-smooth-operator-stineman-1d-interpolation), MATLAB Central File Exchange. Retrieved 11 October 2022.

### 2.4. Statistical Analysis

For statistical HSCORE analyses, the Mann–Whitney nonparametric test was applied for comparisons between two groups. HSCORE data are presented as the median (min. to max.), with *p* ≤ 0.05 considered statistically significant. To characterize mean intensity distribution curves, percentiles were determined. Taking into account that each point of the mean intensity distribution is the mean ± SEM value of the individual distributions corresponding to each patient of the same group, the Mann–Whitney nonparametric test was applied point by point to perform comparisons between the different experimental groups. To establish possible correlations between HSCOREs and computer-assisted image analysis measurements, mean distribution curves were fitted with the Gaussian model, and the amplitude, the mean, and the standard deviation were determined. Statistical analyses were performed using GraphPad Prism Software (GraphPad 8.0, San Diego, CA, USA).

## 3. Results

### 3.1. BCL6 Expression in Different Endometriosis Stages

**Figure 2:**
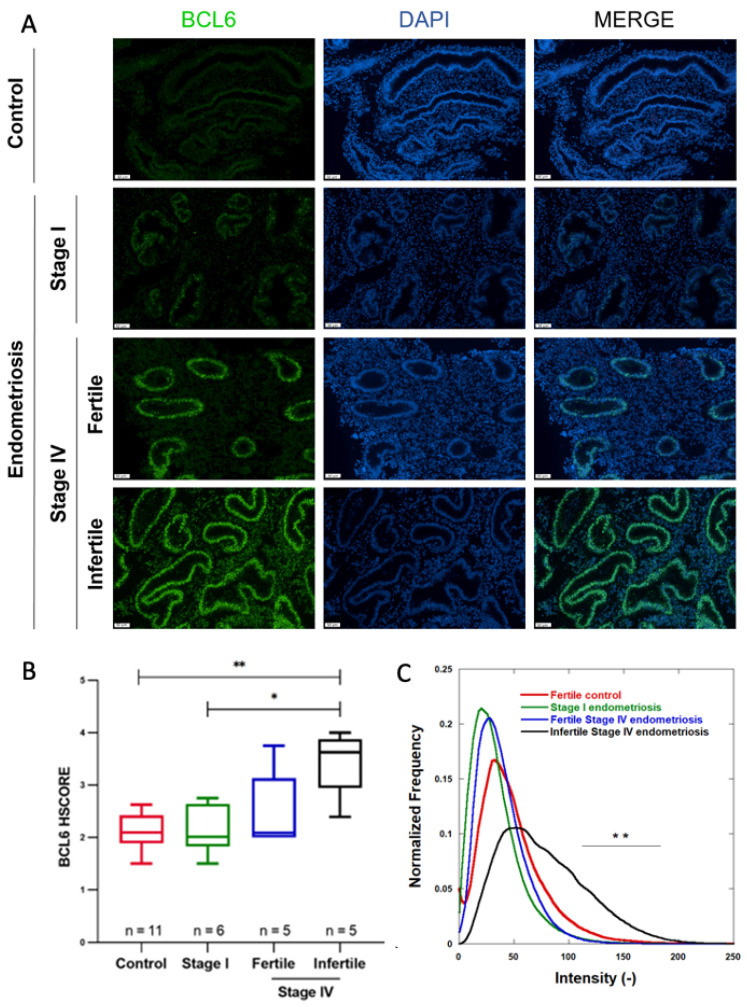
BCL6 expression in endometrium collected during laparoscopy: (**A**) Representative images of BCL6 expression in the endometrium of patients without endometriosis (controls), patients with stage I or stage IV endometriosis with or without associated fertility (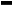 50 µm); (**B**) BCL6 HSCOREs in control patients (*n* = 11) and (**B**) patients with stage I (*n* = 6) or stage IV, fertile (*n* = 5) or infertile (*n* = 5); (**C**) mean normalized BCL6 intensity distributions measured on all scanned sections in each group of patients. * *p* ≤ 0.05, and ** *p* ≤ 0.01.

BCL6 expression was first evaluated by immunofluorescence in the endometrium of a control group and compared to the endometrial expression in women suffering from endometriosis (stage I or stage IV associated with/without infertility, patients from Table 1). Epithelial BCL6 expression levels were found to be comparable in the endometrium of control women and of women with endometriosis stage I. However, higher BCL6 glandular epithelial cell expression was observed in the endometrium of infertile women with stage IV endometriosis (Figure 2A). The HSCORE analysis confirmed those observations. While no difference in BCL6 HSCORE between patients without endometriosis and those with minimal endometriosis (stage I) could be demonstrated (HSCORE 2.095 (*p*25 = 1.890; *p*75 = 2.425) versus 2.012 (*p*25 = 1.825; *p*75 = 2.645), *p* = 0.9829), in the case of severe endometriosis (stage IV) associated with infertility, a higher HSCORE for BCL6 was observed (HSCORE 3.625 (*p*25 = 2.948; *p*75 = 3.875) versus 2.095 (*p*25 = 1.890; *p*75 = 2.425, *p* = 0.0053) (Figure 2B).

A digital computer-assisted quantification of BCL6 was performed on the same sections. BCL6 intensity distributions were calculated in the four different groups of patients (Figure 2C). For the control group, intensities were spread in the range of 11.033–78.448 (Table 3). For women with endometriosis, distributions remained almost within the same ranges (between 4.219 and 56.204 and between 10.819 and 59.447 for stage I and IV endometriosis, respectively). However, in the endometrium of women with stage IV endometriosis associated with infertility, the intensity distribution was spread out in a larger range between 26.138–125.93 (Figure 2C and Table 3), indicating a higher and more heterogeneous BCL6 expression. Statistical analyses evidenced no difference between the intensity distribution of control, stage I, and fertile stage IV endometriosis, whereas a significant difference was observed between those conditions and infertile stage IV endometriosis, mostly in the highest intensity range. 

### 3.2. BCL6 Expression and Fertility Status

**Figure 3:**
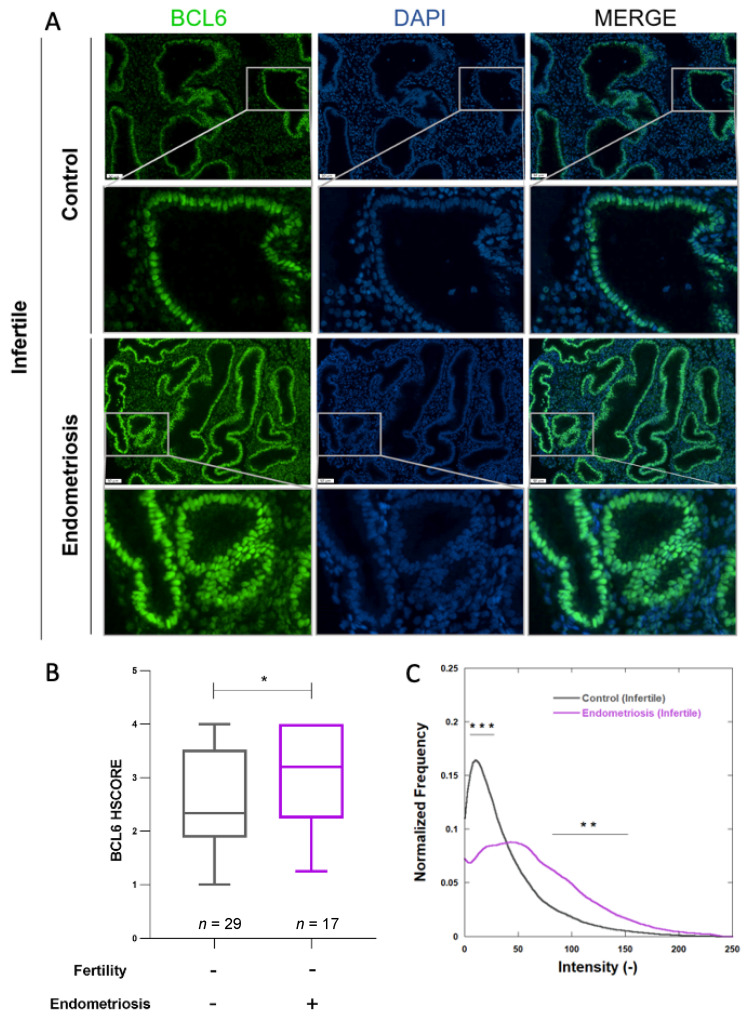
BCL6 expression in the endometrium of infertile patients with and without endometriosis: (**A**) representative images of BCL6 expression (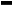 50 µm); (**B**) HSCOREs in the endometrium of infertile women without endometriosis (controls, *n* = 29 and infertile patients with endometriosis (*n* = 17); (**C**) mean normalized BCL6 intensity distributions measured on all scanned sections in each group of patients. * *p* ≤ 0.05, ** *p* ≤ 0.01, and *** *p* ≤ 0.001.

In order to further study the role of BCL6 in endometriosis-related infertility, a comparison of its expression in the endometrium of infertile patients without and with endometriosis was performed on a second set of patients (Table 2). The same methodology was used. In all patients suffering from infertility, clear endometrial BCL6 expression was observed (Figure 3A). HSCORE was indeed the highest in the group of infertile women with endometriosis (HSCORE 2.335 (*p*25 = 1.876; *p*75 = 3.521) versus 3.202 (*p*25 = 2.241; *p*75 = 4.000), *p* = 0.0348) (Figure 3B). When the digital computer-assisted image analysis was used, distribution intensities showed significant differences at low and large intensities (Figure 3C). In fertile or infertile women without endometriosis, BCL6 intensities were spread in a narrow range (see Table 3 and Table 4), with a median at 34.980 (range 11.033–78.448) and at 14.786 (range 0.724–48.51), respectively. The median of BCL6 intensity distribution in the endometrium of infertile women was found at 21.493 in the range of 4.451–54.822. 

The question arises about a possible correlation between the two independent measurements performed on the same samples, i.e., determination of HSCOREs by independent and blinded observers and automatic image analysis.

**Figure 4:**
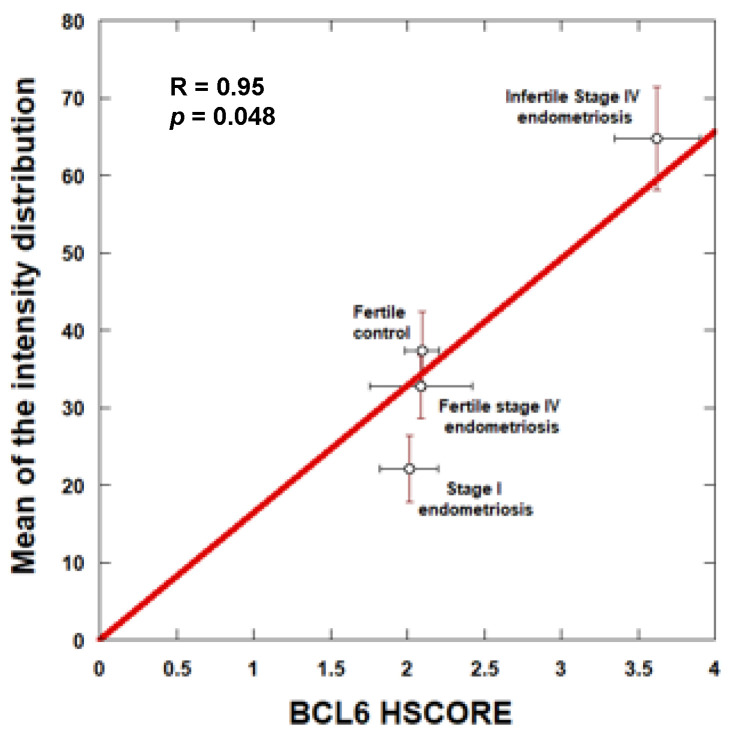
Correlation between computer-assisted image quantification and HSCORE analyses for endometrial BCL6 expression.

The linear regression between the HSCOREs and the mean values of the corresponding intensity distributions showed a significant correlation between the two types of measurements, indicating the robustness of results (Figure 4).

## 4. Discussion

In our study, we confirmed the recently described overexpression of endometrial BCL6 in endometriotic patients by using HSCORE analysis. Indeed, we found that endometrial BCL6 expression was higher in the endometrium of women with stage IV endometriosis. Moreover, the highest HSCORE for BCL6 expression was found in infertile women suffering from endometriosis as compared to infertile women without endometriosis. As previously demonstrated by others, our data suggests a potential role for BCL6 in infertility. Indeed, for up to 50–80% of women having difficulties conceiving, endometriosis has been found to be the main reason for infertility [30,36]. Several hypotheses have been formulated about the way endometriosis impacts fertility. It is known that endometriosis, especially ovarian endometrioma and its surgical treatment, can impair the ovarian reserve. However, this is not the only impact of this pathology on fertility, and one recent theory is linked to progesterone resistance and endometrial receptivity [37]. Endometriosis is clearly associated with a systemic and reversible inflammatory condition that impairs endometrial function [38]. So far, a limited number of studies have investigated the possible involvement of BCL6 in infertility. Interestingly, high levels of endometrial BCL6 in women with unexplained infertility have been recently revealed [1,29] as demonstrated and confirmed in our study by the higher expression of this factor in our infertile group of patients without endometriosis. Increased endometrial BCL6 has been recently considered to be a negative predictor for patients with endometriosis undergoing IVF [3]. Stimulation of pro-inflammatory cytokines by BCL6 in the peritoneal fluid of these women could be responsible for infertility as well as recurrent miscarriages. In addition to its inflammatory power, IL-6 may also be able to directly induce the expression of BCL6. Once transcribed, BCL6, with the help of SIRT1, could inactivate regulators of progesterone but also lead to implantation defects and repeated miscarriages in patients with endometriosis [3].

To date, none of the medical treatments have been able to cure endometriosis, and symptoms recur as soon as the medication is stopped. The development of new biomedical targets, aimed at the cellular and molecular mechanisms responsible for endometriosis, is clearly needed [39]. The use of molecules targeting BCL6 could be a new approach to be investigated in order to treat these numerous patients suffering from endometriosis.

In our study, we used the same semi-quantitative analysis method of BCL6 expression as previously validated [1]. We are perfectly aware that we used the scoring method established for a different experimental method. In the study of Evans-Hoeker et al., negative, medium, or high BCL6 labeling was defined as <1.4, from ≥1.4 to <2.7, and ≥2.7 [1]. Using the cutoff value of 1.4 in HSCORE analysis, BCL6 expression displayed an accuracy of 94% for diagnosing endometriosis. In our study, we used a different antibody for BCL6 detection and performed immunofluorescence for immunostaining. We did not determine HSCORE cutoffs in our setting. However, by using computer-assisted image analysis, a method based on whole pixel quantification and computerization, we were able to clearly demonstrate a good correlation between the two different methods of quantification. The purpose of our work was to compare HSCORE analysis using an automatic quantitative computer-assisted method. It must be noted that the proposed methodology can be implemented with any commercial or free image analysis software as it uses the basic functions in this domain. Moreover, this original computer-assisted method is a robust and reproducible method since it allows one to quantify objectively a large number of endometrial tissue sections.

## 5. Conclusions

By using this original computer-assisted method, our work confirmed that endometrial BCL6 is proportionally expressed with endometriosis stage and that BCL6 overexpression is associated with endometriosis, especially with infertility caused by this gynecological disease. Our quantification method is objective, reliable, robust, and prompt compared to HSCORE analysis.

## Figures and Tables

**Table 1:** Demographics of patients who underwent laparoscopy.

Characteristics	Endometriosis (*n* = 16)	Control (*n* = 11)	*p* Value
**Age (y), mean (SD)**	32.4 ± 6.1	38.4 ± 5.7	0.018
**Body mass index (kg/m^2^), mean (SD)**	22.9 ± 4.5	25.5 ± 4.8	0.112
**Pregnancy history (%)**			
**>1**	3 (18.75)	7 (63.6)	0.042 *
**≤1**	13 (81.25)	4 (26.4)	
**Endometriosis stage, *n* (%)**			
**I**	6 (37.5)		
**IV**	10 (62.5)		

* Chi-squared and Fisher’s exact test analysis; ns = non-significant.

**Table 2:** Demographics of infertile patients with or without endometriosis.

Characteristics	Endometriosis (*n* = 17)	No endometriosis (*n* = 29)	*p* Value
**Age (y), mean (SD)**	34.5 ± 5.0	36.2 ± 3.4	0.244
**Body mass index (kg/m^2^), mean (SD)**	22.0 ± 7.6	26.04 ± 5.8	0.166
**Infertility type, *n* (%)**			
**Primary**	6 (35.3)	13 (44.8)	0.565 *
**Secondary**	11 (64.7)	15 (51.7)	
**Not available**	0 (0)	1 (3.45)	
**Previous miscarriages, *n* (%)**			
**>1**	10 (58.8)	16 (55.2)	0.774 *
**≤1**	7 (41.2)	13 (44.8)	

* Chi-squared and Fisher’s exact test analysis; ns = non-significant.

**Table 3:** Percentiles of BCL6 intensity and distribution in control, endometriosis (Stage I and IV), and infertility-associated endometriosis stage IV patients.

Percentiles	Fertile Control	Fertile Stage I	Fertile Stage IV	Infertile Stage IV
10	11.033	4.2194	10.819	26.138
25	22.021	11.996	16.810	40.352
50	34.980	21.942	27.304	64.295
75	54.108	36.508	42.114	95.765
90	78.448	56.204	59.477	125.93

**Table 4:** Percentiles of BCL6 intensity and distribution in infertile patients without or with associated endometriosis.

Percentiles	Infertile Control	Infertile with Endometriosis
10	0.724	4.4512
25	4.912	11.464
50	14.786	21.493
75	28.596	34.918
90	48.512	54.822

## Data Availability

Not applicable.
